# Functional Analysis of TRPA1, TRPM3, and TRPV1 Channels in Human Dermal Arteries and Their Role in Vascular Modulation

**DOI:** 10.3390/ph17020156

**Published:** 2024-01-25

**Authors:** Eduardo Rivera-Mancilla, Linda Al-Hassany, Heleen Marynissen, Dorien Bamps, Ingrid M. Garrelds, Jérôme Cornette, A. H. Jan Danser, Carlos M. Villalón, Jan N. de Hoon, Antoinette MaassenVanDenBrink

**Affiliations:** 1 Division of Vascular Medicine and Pharmacology, Department of Internal Medicine, Erasmus University Medical Center Rotterdam, P.O. Box 2040, 3000 CA Rotterdam, The Netherlands; e.riveramancilla@erasmusmc.nl (E.R.-M.); l.alhassany@erasmusmc.nl (L.A.-H.); i.vandenberg-garrelds@erasmusmc.nl (I.M.G.); a.danser@erasmusmc.nl (A.H.J.D.); 2 Department of Pharmaceutical and Pharmacological Sciences, Center for Clinical Pharmacology, KU Leuven, 300 Leuven, Belgium; heleen.marynissen@uzleuven.be (H.M.); dorien.bamps@kuleuven.be (D.B.); jan.dehoon@uzleuven.be (J.N.d.H.); 3 Department of Obstetrics and Fetal Medicine, Erasmus University Medical Center Rotterdam, P.O. Box 2040, 3000 CA Rotterdam, The Netherlands; j.cornette@erasmusmc.nl; 4 Department of Pharmacobiology, Cinvestav-Coapa, Mexico City C.P. 14330, Mexico; cvillalon@cinvestav.mx

**Keywords:** CGRP, human dermal artery, TRP channels, pharmacological mechanisms, vascular tone, vasodilation

## Abstract

Transient receptor potential (TRP) channels are pivotal in modulating vascular functions. In fact, topical application of cinnamaldehyde or capsaicin (TRPA1 and TRPV1 channel agonists, respectively) induces “local” changes in blood flow by releasing vasodilator neuropeptides. We investigated TRP channels’ contributions and the pharmacological mechanisms driving vasodilation in human isolated dermal arteries. Ex vivo studies assessed the vascular function of artery segments and analyzed the effects of different compounds. Concentration–response curves to cinnamaldehyde, pregnenolone sulfate (PregS, TRPM3 agonist), and capsaicin were constructed to evaluate the effect of the antagonists HC030031 (TRPA1); isosakuranetin (TRPM3); and capsazepine (TRPV1). Additionally, the antagonists/inhibitors olcegepant (CGRP receptor); L-NAME (nitric oxide synthase); indomethacin (cyclooxygenase); TRAM-34 plus apamin (K^+^ channels); and MK-801 (NMDA receptors, only for PregS) were used. Moreover, CGRP release was assessed in the organ bath fluid post-agonist-exposure. In dermal arteries, cinnamaldehyde- and capsaicin-induced relaxation remained unchanged after the aforementioned antagonists, while PregS-induced relaxation was significantly inhibited by isosakuranetin, L-NAME and MK-801. Furthermore, there was a significant increase in CGRP levels post-agonist-exposure. In our experimental model, TRPA1 and TRPV1 channels seem not to be involved in cinnamaldehyde- or capsaicin-induced relaxation, respectively, whereas TRPM3 channels contribute to PregS-induced relaxation, possibly via CGRP-independent mechanisms.

## 1. Introduction

The superfamily of transient receptor potential (TRP) channels comprises six subfamilies, including the ankyrin (TRPA1); the melastatin (TRPM1-7); the vanilloid (TRPV1-6); the canonical (TRPC1-7); the mucolipin (TRPML1-3); and the polycystin (TRPP1-3, PKD2, and PKD2-L2) subfamilies [1]. Overall, TRP channels modulate the influx of mainly Ca^2+^ (with the exceptions of TRPM4 and TRPM5) and Mg^2+^ across the plasma membrane [2], which is essential for maintaining homeostasis in several physiological processes, including taste perception, thermosensation, female reproduction, and nociception [2,3,4].

Besides being implicated in nociception and pain [5], three of these subfamilies, namely, the TRPA1, TRPM3, and TRPV1 channels, have been suggested to be involved in the modulation of vascular tone [5,6,7,8], suggesting a potential role as therapeutic targets for managing pain and vascular-related disorders. In fact, these channels may be expressed in vascular smooth muscle—and/or endothelial—cells [6,8,9], modulating vascular responses through increased intracellular Ca^2+^ levels, depolarization of membrane potential, triggering second messenger pathways, or via the stimulation of endothelial nitric oxide synthase and prostacyclin synthesis [3,6,7,8,9]. Moreover, these channels are expressed in perivascular sensory nerves [3,6,7,8], where they directly or indirectly modulate vascular tone by releasing calcitonin gene-related peptides (CGRPs) or substance P [6,10,11].

In this respect, the activation of TRPA1 (e.g., by cinnamaldehyde) [12], TRPM3 (e.g., by pregnenolone sulfate [PregS] or CIM 0216) [6,13], or TRPV1 (e.g., by capsaicin) [10,11] channels induces vasodilatory responses in a wide variety of isolated blood vessels through different mechanisms, such as the release of nitric oxide from endothelial cells and/or the release of CGRP from perivascular sensory nerves [11,12,13,14,15]. These include human and porcine coronary arteries [10,16]; human skeletal muscle feed arteries [17]; rat mesenteric [6], aorta [18,19], and cerebral arteries [20]; and human [14,21,22,23] and animal [12,15] in vivo models with the topical application of capsaicin or cinnamaldehyde. Moreover, knockout animal models of TRPA1 [24,25], TRPM3 [6,26], and TRPV1 [27,28] have demonstrated changes and/or “alterations” to the baseline vascular tone, suggesting an important function of these channels in modulating vascular responses.

Notwithstanding the evidence on the vasoactive role of TRP channels in both in vitro [6,10,16,19,20] and in vivo [12,15,29] models, there is a controversy considering that activation of TRPV1 channels by capsaicin [15] or TRPM3 channels by PregS [6,26] can induce both vasodilation and vasoconstriction, depending on the vascular bed, the species, and the experimental protocols. The exact mechanism(s) by which TRPA1, TRPM3, and TRPV1 channels modulate vascular tone remains uncertain. Therefore, to elucidate how TRP channels contribute to the modulation of vascular tone, we evaluated the vasoactive effects of the agonists cinnamaldehyde, PregS, and capsaicin in human isolated dermal arteries (i.e., resistance arteries) from subcutaneous fat obtained from normotensive pregnant women, who present an enhanced endothelium-dependent vascular response [30]. Furthermore, subcutaneous fat can be easily removed from pregnant women during a caesarean section without additional burden, creating a unique model to directly assess the vascular function, as opposed to in vivo models, where vascular responses are assessed at superficial skin levels. Therefore, using human isolated dermal arteries enables the direct exploration of TRP channel function in a context closely resembling human physiology. This approach may contribute to a better understanding of the mechanisms underlying the modulation of the vascular tone and provide new insights for potential therapeutic targets. In addition, we pharmacologically investigated the mechanisms involved in these effects using different antagonists and/or blockers (Table S1), considering their locations within the vessel wall. Moreover, since the activation of the TRPA1, TRPM3, and TRPV1 channels induces CGRP release [11,12,13,14,15], we also analyzed the agonist-induced CGRP release in organ bath fluids.

Our findings suggest that, in this experimental model, TRPA1 and TRPV1 channels are not implicated in cinnamaldehyde- or capsaicin-induced relaxation, respectively. Conversely, TRPM3 channels play a role in PregS-induced relaxation, likely mediated by CGRP-independent mechanisms.

## 2. Results

### 2.1. Activation of TRPM3, but Not TRPA1 or TRPV1, Channels Modulates Vasodilation in Human Isolated Dermal Arteries

In organ baths, we assessed vascular function and explored the pharmacological mechanisms of TRP agonists in dermal artery segments. Concentration-dependent relaxant responses were observed after administration of cinnamaldehyde, PregS, and capsaicin. The maximum relaxant responses (E_max_) produced at the highest concentration of cinnamaldehyde (1 mM), PregS (100 µM), and capsaicin (100 µM) and their potencies (expressed as pEC_50_) were E_max_ = 74 ± 11%, pEC_50_ ≤ 3.57 ± 0.33; E_max_ = 40 ± 3%, pEC_50_ ≤ 4.69 ± 0.58 and E_max_ = 87 ± 4%, and pEC_50_ ≤ 4.74 ± 0.10, respectively (Figure S1, Table S2). The vehicle of cinnamaldehyde and PregS (DMSO) or capsaicin (ethanol) did not produce any effect. Finally, the analysis of endothelial function resulted in a mean dilation response of 84 ± 14% (induced by 10 nM substance P) of the precontraction induced by 10 nM U46619.

**Figure 1:**
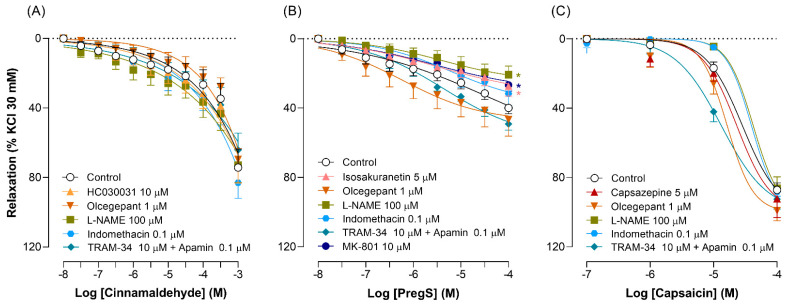
Effect of different compounds on the vasodilatory responses induced by TRP channel agonists in human isolated dermal arteries. Relaxation responses to (**A**) cinnamaldehyde (0.01 µM-1 mM, *n* = 6–8), (**B**) PregS (0.01–100 µM, *n* = 8–22), and (**C**) capsaicin (0.1–100 µM, *n* = 8–19), in the absence (control) or presence of the TRP antagonists (**A**) HC030031 (10 µM), (**B**) isosakuranetin (5 µM), and (**C**) capsazepine (5 µM), as well as in the presence of different receptor blockers and enzyme inhibitors: olcegepant (1 µM), L-NAME (100 µM), indomethacin (0.1 µM), TRAM-34 (10 µM) plus apamin (0.1 µM), and MK-801 (10 µM, only for PregS). Data are expressed as mean  ±  SEM. * *p* < 0.05 versus control.

Furthermore, we evaluated the effects of several compounds (see Table S1) on the relaxant responses induced by TRP channel agonists. The vasorelaxant responses and E_max_ induced by cinnamaldehyde (E_max_ 74 ± 11%) and capsaicin (E_max_ 87 ± 4%) remained unchanged (*p* > 0.05) after exposure to (i) 10 µM HC030031 (E_max_ 73 ± 10%) or 5 µM capsazepine (E_max_ 92 ± 11%), respectively (Figure 1A,C). Similarly, olcegepant (1 µM), L-NAME (100 µM), indomethacin (0.1 µM), and TRAM-34 (100 µM) plus apamin (0.1 µM) did not affect the relaxation responses induced by cinnamaldehyde or capsaicin (Figure 1A,C).

Conversely, the vasorelaxant responses to PregS, along with the maximal response at the highest concentration of PregS (100 µM, E_max_ 40 ± 3%), were (i) significantly (*p* < 0.05) inhibited by 5 µM isosakuranetin (E_max_ 27 ± 6%), 100 µM L-NAME (E_max_ 21 ± 5%), and 10 µM MK-801 (E_max_ 27 ± 4); and (ii) remained unaffected (*p* > 0.05) by olcegepant (1 µM), indomethacin (0.1 µM) and TRAM-34 (100 µM) plus apamin (0.1 µM) (Figure 1B).

### 2.2. CGRP-Like Immunoreactivity Levels in the Organ Bath Fluid Post-Agonist-Exposure

**Figure 2:**
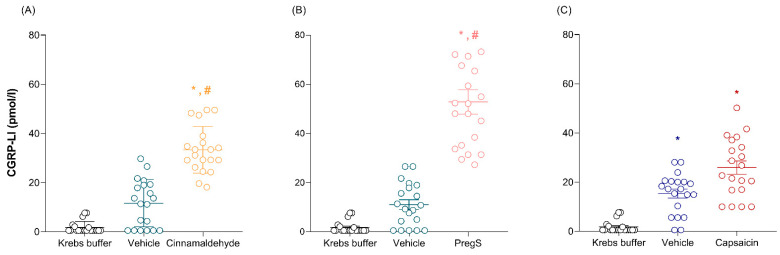
Activation of TRPA1, TRPM3, and TRPV1 channels induces the release of CGRP. CGRP-LI levels were measured in organ bath fluids after exposure to (**A**) cinnamaldehyde, (**B**) PregS, and (**C**) capsaicin, or vehicles (DMSO (vehicle of cinnamaldehyde and PregS) or ethanol (vehicle of capsaicin)) in human dermal artery segments. Data are expressed as mean  ±  SEM in pmol/L. * *p* < 0.05 versus Krebs buffer (control group). ^#^ *p* <  0.05 versus the vehicle. The values are representative of 20 patients.

Cinnamaldehyde, PregS, and capsaicin induced a significant (*p* < 0.05) increase in CGRP-like immunoreactivity (CGRP-LI) release compared to the levels observed in Krebs buffer (1.8 ± 1.0 pmol/L, below the detection range): (i) cinnamaldehyde: 33.0 ± 2.0 pmol/L (Figure 2A); (ii) PregS: 52.0 ± 5.0 pmol/L (Figure 2B); and (iii) capsaicin: 26.0 ± 3.0 pmol/L (Figure 2C). Notably, exposure to DMSO (vehicle of cinnamaldehyde and PregS) exhibited no significant increase in CGRP release (*p* = 0.0602 and *p* = 0.0528), i.e., 12.0 ± 2.0 pmol/L and 11.0 ± 2.0 pmol/L, respectively (Figure 2A,B), compared to the control (Krebs buffer). In contrast, ethanol (vehicle of capsaicin) induced a significant (*p* = 0.0003) increase in CGRP release (15.0 ± 2.0 pmol/L) compared to Krebs buffer, but not when compared to that induced by capsaicin (Figure 2C).

### 2.3. Localization of TRP Channels in Human Dermal Arteries

Immunofluorescence microscopy was used to visualize the localization of the TRPA1, TRPM3, and TRPV1 channels in the intact vessel wall. The immunostaining was performed using antibodies with confirmed specificity against TRPA1, TRPM3, or TRPV1 in different tissues and/or cells, including dorsal root and retinal ganglia, optic nerve oligodendrocytes, and human stem-cell-derived sensory neurons [31,32,33,34]. Moreover, negative controls were utilized by omitting the primary antibody to ensure the specificity of these antibodies in our preparation (see Figure S2). Nevertheless, it is important to emphasize that the specificities of some anti-TRP antibodies have not been fully standardized in human cells [34].

**Figure 3:**
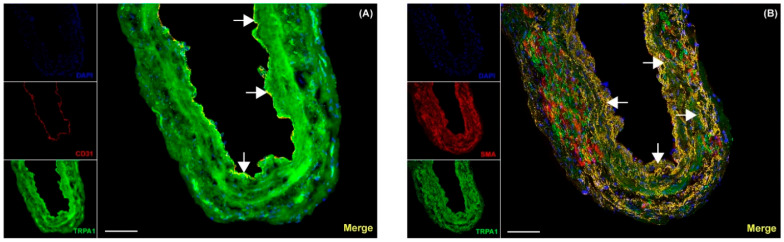

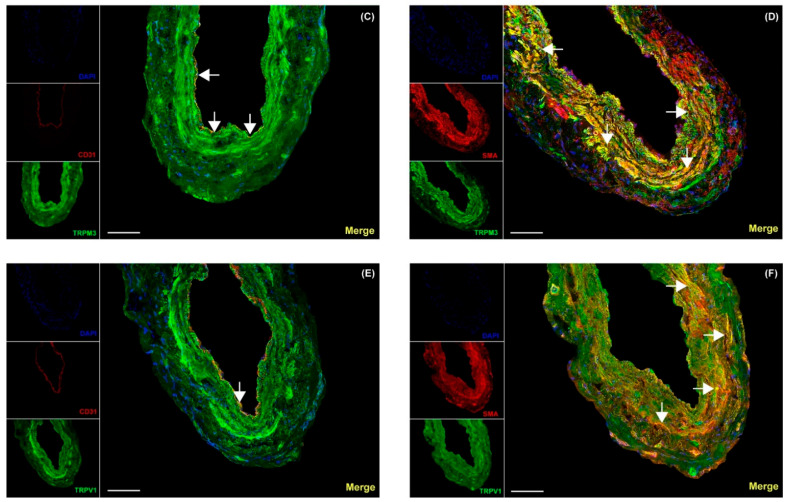
Locations of TRPA1, TRPM3, and TRPV1 channels in human dermal arteries. Confocal microscopy images of intact human dermal arteries labeled with antibodies against (**A**,**C**,**E**) CD31 endothelial cells or (**B**,**D**,**F**) actin α-smooth muscle cells (red); (**A**,**B**) TRPA1; (**C**,**D**) TRPM3; (**E**,**F**) TRPV1; and DAPI for nuclear staining (blue). Arrows represent colocalization of TRP channels in endothelial and smooth muscle cells (yellow). The images are representative of 3 independent patients. Negative controls are shown in Figure S2. Scale bars represent 170 µM.

**Figure 4:**
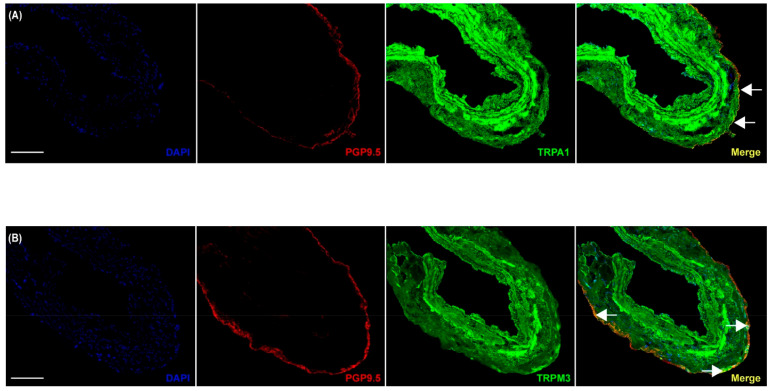

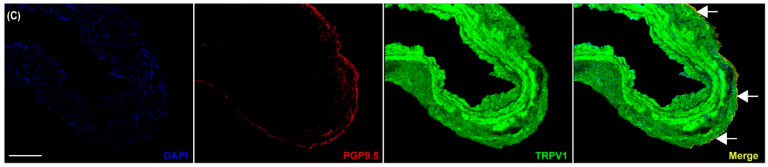
TRPA1, TRPM3, and TRPV1 channels are located in perivascular nerve endings. Confocal microscopy images of intact human dermal arteries labeled with PGP9.5 (marker of perivascular nerve endings, red) or (**A**) TRPA1; (**B**) TRPM3; and (**C**) TRPV1 (green) antibodies; as well as with DAPI for nuclear staining (blue). Arrows represent colocalization of TRP channels in perivascular nerve endings (yellow). The images are representative of 3 independent patients. Scale bars represent 170 µM.

As illustrated in Figure 3, it appears that the TRPA1 (B), TRPM3 (D), and TRPV1 (F) channels are mainly located in smooth muscle cells (in the intima or media layers), and apparently, to a lesser extent, in endothelial cells (Figure 3A,C,E). Furthermore, immunostaining with the neuronal marker PGP9.5 demonstrated the presence of these channels in perivascular sensory nerves innervating the adventitial layers of dermal arteries (Figure 4).

## 3. Discussion

Both in vitro and in vivo studies have investigated the mechanisms involved in the vasodilatory effects induced by cinnamaldehyde, PregS, and capsaicin [6,10,16,19,20], which can include, among others, the activation of the nitric oxide pathway or K^+^ channels, the inhibition of Ca^2+^ influx, and/or the induction of CGRP release from perivascular nerves [6,10,16,19,20]. However, these mechanisms, at least in part, are controversial, suggesting that they may depend on the vascular bed and/or on the species under study.

Our findings in human isolated dermal arteries show that cinnamaldehyde- and capsaicin-induced relaxations are not mediated by, respectively, TRPA1 and TRPV1 channels and/or the activation of CGRP receptor (Figure 1A,C). Moreover, the vasodilation induced by cinnamaldehyde and capsaicin appears to implicate non-specific mechanisms, as these responses were not affected by any of the inhibitors/blockers we used. In contrast, the vasodilatory responses and the E_max_ induced by PregS were significantly inhibited by 5 µM isosakuranetin, 100 µM L-NAME, and 10 µM MK-801 (Figure 1B), suggesting that: (i) TRPM3 channels are involved in PregS-induced relaxations and (ii) these responses may be mediated by mechanisms including the activation of NMDA receptors and/or the nitric oxide pathway. Admittedly, these suggestions are based on the assumption that: (i) cinnamaldehyde, PregS, and capsaicin are relatively selective agonists that can bind to their respective ion channels (pEC_50_ values: cinnamaldehyde = 4.2, PregS = 4.9, capsaicin = 7.5) [35,36,37], inducing their activation and, consequently, vasorelaxation (or changes in blood flow) [6,12,14,19,22,23]; and/or, (ii) as described in in vivo models, the same agonists can trigger the activation of TRP channels located at the nerve endings of sensory nerves [23], which innervate resistance arteries. It is important to mention that, although the potencies to induce vasodilation of human dermal arteries are similar (*p* > 0.05) for cinnamaldehyde (pEC_50_ ≤ 3.57 ± 0.33), PregS (pEC_50_ ≤ 4.69 ± 0.58), and capsaicin (pEC_50_ ≤ 4.74 ± 0.10), these agonists elicit different relaxation responses (see Figure S1). Nevertheless, since the pEC_50_ values were calculated at the highest feasible concentration of the agonists and the concentration–response curves did not necessarily reach the maximum response, direct comparisons of the potencies may not be realistic and could be misleading.

### 3.1. Vasoactive Role of TRPA1 and TRPV1 Channels

Both cinnamaldehyde and capsaicin induced concentration-dependent relaxations (Figure 1A,C). To investigate the mechanism(s) involved, we evaluated the effects of “selective” antagonists for TRPA1 and TRPV1 channels, as well as different blockers of pathways that could be implicated.

Our results on the vasodilatory effects of cinnamaldehyde align with previously reported studies that evaluated cinnamaldehyde-induced responses in isolated porcine coronary arteries [16], rat aortas [18] or cerebral arteries [20], and ventricular cardiomyocytes [19], or in an in vivo model by the topical application of cinnamaldehyde [12,22]. As the vasodilation induced by cinnamaldehyde was not modified by 10 µM HC030031 or 1 µM olcegepant, these responses do not appear to be mediated by the activation of TRPA1 channels or CGRP receptors (Figure 1A). This implies that the involvement of additional mechanisms, potentially linked to the inhibition of L-type Ca^2+^ channels [18,19], might be implicated. Furthermore, our results suggest that the relaxations to cinnamaldehyde are endothelium-independent and not mediated by the cyclooxygenase pathway, as they were resistant to blockades by 100 µM L-NAME (nitric oxide synthase inhibitor) and 0.1 µM indomethacin (cyclooxygenase inhibitor), respectively (Figure 1A). These results are consistent with other previous studies reporting that TRPA1 activation induces vasodilation through nitric oxide- and/or cyclooxygenase-independent mechanisms in porcine coronary [16] and rat cerebral [20] arteries.

Moreover, although Ca^2+^-activated K^+^ channels are expressed in endothelial cells [38], and the K^+^ channels blockers apamin and TRAM-34 abolish cinnamaldehyde-induced relaxation responses in cerebral arteries [20], these responses were resistant to an apamin plus TRAM-34 blockade in our study (Figure 1A), even when using a 10-fold higher concentration of TRAM-34 than that reported earlier [20]. It is also important to note that: (i) these authors used a different TRPA1 channel agonist (i.e., allyl isothiocyanate) and evaluated its vasodilator effect in pressurized arteries at 70 mmHg, as opposed to our isometric tension measurements [20].

Likewise, the vasorelaxant responses to capsaicin were capsazepine- and olcegepant-insensitive (Figure 1C), suggesting that these responses are not mediated by the activation of TRPV1 channels or via the release of CGRP. In agreement with our findings, Gupta et al. [10] and Fujimoto and Mori [39] reported that capsaicin-induced relaxation responses in human and porcine distal coronary arteries [10] or in the rat ileum [39] are not mediated by CGRP receptors or TRPV1 channels. Similarly, our results with L-NAME, indomethacin, and the combination of TRAM-34 plus apamin suggest that the nitric oxide pathway and/or the small- and/or intermediate-conductance Ca^2+^-activated K^+^ channels are not responsible for capsaicin-induced relaxation (Figure 1C), as previously reported [10]. Evidently, additional mechanisms are implicated in capsaicin-induced vasodilatory responses in resistance arteries. In fact, it has been suggested that the activation of cannabinoid CB_1_ receptors may play an important role in the mechanisms of action of capsaicin in modulating vasorelaxation [40].

In our study, it seems that the relaxations to cinnamaldehyde and capsaicin involve non-specific mechanisms, not including activation of TRPA1 or TRPV1 channels or CGRP receptors. Considering that in in vivo models, as well as in some vascular beds, these responses are mediated by TRPA1 channels or CGRP receptors [11,12,14,21,41], it appears that the mechanisms involved depend on the vascular beds and experimental methods. For example, the EC_50_ of allyl isothiocyanate (16.4 µM) [20] was lower than that of cinnamaldehyde in our experiments to induce relaxation (~357 µM), which might suggest that cinnamaldehyde possesses non-specificity and/or lower potency, at least in our model, to activate TRPA1 channels. Furthermore, it is important to note that our findings cannot be directly compared with those in vivo models in which changes in dermal blood flow induced by the topical application of cinnamaldehyde or capsaicin are typically measured using laser Doppler perfusion imaging. This non-invasive model assesses the effects of agonists at superficial skin levels, likely acting on TRP channels located in sensory nerves that innervate the skin and/or blood vessels [12,14,21,22,23]. In contrast, we studied arteries from deeper dermal layers, allowing us to assess the vascular function and/or the effects of agonists at the vascular level.

In addition, it is known that TRPA1 and TRPV1 channels are expressed in endothelial and smooth muscle cells, as well as in perivascular nerves [7,8,9], where they modulate vascular tone. Confocal microscopy shows that TRPA1 and TRPV1 channels appear to be mainly located in smooth muscle cells (Figure 3B,F) and, apparently, to a lesser extent, in endothelial cells (Figure 3A,E) and perivascular nerve endings (Figure 4A,C). Certainly, the activation of TRPV1 channels in smooth muscle cells induces constriction [8,9]; however, it has been demonstrated that capsaicin can also induce relaxant responses in smooth muscle cells via the modulation of Ca^2+^ influx [40,42]. Nevertheless, since our results show that the relaxation responses involve TRPA1- and TRPV1-independent mechanisms (Figure 1), we can suggest that: (i) TRPA1 and TRPV1 channels have no canonical activity in smooth muscle cells, which might explain the relaxant responses; and (ii) cinnamaldehyde [19] and capsaicin [42] can only act on large-conductance Ca^2+^-activated K^+^ channels, but not on small- or intermediate-conductance Ca^2+^-activated (Figure 1A,C) channels, by inhibiting Ca^2+^ influx and inducing vasorelaxation.

### 3.2. Role of TRPM3 Channels in the Modulation of the Vascular Tone

The vasoactive role of TRPM3 channels has been controversial, as PregS can induce both vasoconstriction [26] and vasodilation [6]. Our results in human isolated dermal arteries show that PregS induces concentration-dependent vasodilation, which is mediated by the activation of TRPM3 channels. This finding is supported considering that the vasodilatory responses induced by PregS, as well the E_max_ to 100 µM PregS, were significantly inhibited by 5 µM isosakuranetin (Figure 2B), which is consistent with earlier studies [6].

To confirm our findings, we also analyzed the effect of the TRPM3 channel inhibitor 2-APB (75 µM) [26] on PregS-induced relaxation, as well as the effect of isosakuranetin on the synthetic TRPM3 channel agonist, CIM 0216 [6] (Figure S3). Therefore, we suggest that TRPM3 channels can modulate vascular tone in human dermal arteries, probably attributed, at least in part, to endothelium-dependent mechanisms considering that 100 µM L-NAME produced a significant blockade of PregS-induced vasorelaxation (Figure 2B), and that TRPM3 channels are located, albeit to a lesser extent, in endothelial cells (Figure 3C).

In addition, immunofluorescence microscopy showed that TRPM3 channels are also located in smooth muscle cells (Figure 3D), which is in line with earlier studies, where they were found to be involved in inducing contractile responses [26] and/or lacked functionality [6]. Certainly, we have no evidence that PregS-induced vasodilation is mediated by TRPM3 channels located in smooth muscle cells, which contrasts with some previous findings [26]. However, it is important to emphasize that: (i) some TRP channels, including the TRPM subfamily, exhibit constitutive activity when expressed in tissue-cultured cells, but not in intact tissue [43], potentially related to Ca^2+^ influx producing vasoactive effects; and (ii) although there is no evidence that PregS is involved in the inhibition of Ca^2+^ influx in smooth muscle cells, progesterone may inhibit PregS-evoked Ca^2+^ signaling [44]. Accordingly, progesterone has an important biological effect during reproduction [44], when its levels are high (e.g., during pregnancy). While we did not measure progesterone levels in our study, we hypothesize that the vasodilator effect mediated by TRPM3 channels could be due to the potent effect of progesterone by inhibiting Ca^2+^ entry, as our tissues were obtained from pregnant women.

Few studies have described the mechanism(s) involved in the vasoactive effect of TRPM3 channels [6,26]. Since PregS-induced relaxation responses were olcegepant-, indomethacin-, or TRAM-34-plus-apamin-insensitive, our results suggest that these responses are mediated by additional mechanisms unrelated to the activation of CGRP receptors, K^+^ channels, or the prostaglandin pathway (see Figure 1B). In contrast, others have reported that the relaxations due to PregS were partially inhibited by K^+^ channel blockers and by olcegepant in endothelium-denuded mesenteric arteries containing perivascular nerves [6], suggesting differences between vascular beds or a dependence on the presence of a functional endothelium or perivascular nerves.

Finally, since PregS has been reported to be a positive allosteric modulator of the NMDA receptors [45], we also evaluated the effect of MK-801 (an NMDA receptor antagonist) on PregS-induced relaxation. The fact that the vasodilatory response to PregS was inhibited by 10 µM MK-801 (Figure 2B) suggests that NMDA receptors modulate the function of TRPM3 channels. Although the involvement of NMDA receptors was not analyzed in mouse mesenteric arteries [6], it has been reported that the activation of NMDA receptors is involved in the inhibition of vasodilation induced by electrical stimulation; this is attributed to the inhibition of the neurogenic release of CGRP via a blockade of NMDA receptors [46]. Therefore, in addition to their excitatory function in the central nervous system, NMDA receptors might play an important role in the modulation of vascular tone, which would represent a potential therapeutic target in vascular diseases. However, as in our current experiments the response to PregS seemed to be, at least in part, unrelated to CGRP release; the effect of NMDA receptors on vascular tone modulation is most likely due to direct vascular mechanisms. Clearly, further experiments which fall beyond the scope of the current study are required in order to investigate the mechanism(s) involved.

### 3.3. CGRP Release via the Activation of TRP Channels

CGRP release upon the activation of TRPA1, TRPM3, and TRPV1 channels has been previously reported [10,11,12,13,14,15,47]. We observed that, after exposure to cinnamaldehyde, PregS, or capsaicin, there was a significant increase in CGRP release from the human dermal artery segments (~1.5 mg tissue in 6 mL organ bath fluid) when compared with that induced by Krebs buffer or the vehicles of cinnamaldehyde (Figure 2A) or PregS (Figure 2B). Notably, exposure to DMSO showed a clear-cut tendency with the increase in CGRP levels (Figure 2A,B); however, the results were not statistically significant, and there is evidence that DMSO does not have an effect on CGRP release, per se [48]. Moreover, a similar increase was observed after using the vehicle of capsaicin (i.e., ethanol, Figure 2C), confirming that both capsaicin and ethanol can activate TRPV1 channels [10].

Paradoxically, the relaxations induced by the same agonists are not mediated by CGRP receptors, as they were not affected by olcegepant (Figure 1). A possible explanation for these findings could be related to: (i) the total concentration of CGRP released and (ii) the fragmentation of CGRP due to the lack of protease inhibitors, and then cross-reactivity with CGRP fragments or another ligand, but not CGRP. Firstly, the local CGRP concentrations in the organ bath fluids (i.e., cinnamaldehyde: ~33.0 pM; PregS: ~52.0 pM; and capsaicin: ~26.0 pM) should be at least 10,000 times higher in the artery segments, as we mounted ~1.5 mg tissue in 6 mL of organ bath fluid; thus, they should be in the nanomolar range. Secondly, CGRP is a peptide with a short half-life that can be quickly degraded by proteases [49]. Therefore, under physiological conditions, CGRP release occurs at a local level between sensory nerve terminals and the layers of the vascular smooth muscle. Indeed, we collected the fluids from the organ baths immediately after completing the concentration–response curves for the TRP channel agonists and mixed them with protease inhibitors (i.e., aprotinin) to prevent CGRP degradation. Nonetheless, we cannot categorically confirm that its degradation was interrupted and/or that the CGRP released in our preparation was diluted.

## 4. Materials and Methods

### 4.1. Inclusion and Exclusion Criteria

Normotensive pregnant women ≥ 18 years of age, who had blood pressure values (systolic/diastolic) of ≤139/89 mmHg and were undergoing elective caesarean section, were included after receiving informed consent. Patients with admission to an intensive care unit, subjects who underwent an emergency caesarean section, or those who were unable to provide informed consent were excluded.

### 4.2. Human Tissues

In total, 43 normotensive pregnant women (77% Caucasian, 23% other ethnicities (African: 9%; Latin American: 2%; other: 12%)) with a median (interquartile range) age of 34 (30–36) years, gestational age of 39 (38–40) weeks, and blood pressure values (systolic/diastolic) of 119 (109−129)/72 (65–80) mmHg were included. Dermal arteries were isolated from a 1.5 cm piece of deep subcutaneous fat tissue, which was removed from an area just proximal to the rectus sheet, from normotensive pregnant women undergoing caesarean section at the Department of Obstetrics and Fetal Medicine at Erasmus MC, Rotterdam, The Netherlands.

### 4.3. Functional Ex Vivo Studies (Wire Myography Experiments)

After removal, tissues were placed in cold medium M199 (Gibco, Invitrogen, Carlsbad, CA, USA) and immediately transported to the laboratory. The dermal arteries (internal diameter 150–250 µm) were isolated from the fat tissue, dissected, cut into small segments of about 1.5–2 mm each, and mounted in Mulvany myographs (ADinstruments, Danish Myograph Technology, Aarhus, Denmark) containing oxygenated Krebs bicarbonate solution at 37 °C (see Supplemental Methods, “S1.1 Wire-myography experiments”, for details). Concentration–response curves of vehicles (i.e., DMSO (vehicle of cinnamaldehyde and PregS), ethanol (vehicle of capsaicin)) or the agonists, i.e., cinnamaldehyde (TRPA1, 0.01 µM–1 mM) [16,18], PregS (TRPM3, 0.01–100 µM) [13], and capsaicin (TRPV1, 0.1–100 µM) [10] were constructed in a parallel design, in the absence or presence of the antagonists HC030031 (TRPA1, 10 µM) [16], isosakuranetin (TRPM3, 5 µM) [13], and capsazepine (TRPV1, 5 µM) [10,39,50] (Table S1). Before constructing the concentration–response curves of the agonists, precontraction was induced using KCl 30 mM, as previously reported [10]. After the concentration–response curves, a single concentration of 10 nM substance *p* was administered after precontraction with U46619 (10 nM) to assess endothelial functionality [10].

Additionally, to evaluate the possible mediator(s) involved in the agonist-induced relaxation, different pharmacological tools were applied (Table S1), including: (i) 1 µM olcegepant (CGRP receptor antagonist) [6,10]; (ii) 100 µM L-NAME (nitric oxide synthase inhibitor) [10,16]; (iii) 0.1 µM indomethacin (cyclooxygenase inhibitor) [10]; and (iv) 100 µM TRAM34 plus 0.1 µM apamin (K^+^ channel blockers) [20]. Moreover, since PregS can activate and modulate NMDA receptors [45], we also investigated the role of NMDA receptors in PregS-induced relaxation using the NMDA receptor antagonist MK-801 (10 µM) [51].

### 4.4. Immunofluorescence Microscopy

Intact dermal arteries were used to visualize the localization of the TRPA1, TRPM3, and TRPV1 channels (see Supplemental Methods, “S1.2 Immunofluorescence microscopy”, for details). Tissue segments were fixed with 4% paraformaldehyde for 10 min, permeabilized in PBS/0.2% Triton X-100 for 10 min, and blocked for 2 h with blocking buffer. Then, tissues were incubated overnight at 4 °C with a primary antibody against TRPA1, TRPM3, or TRPV1 channels in combination with the markers for endothelial cells, smooth muscle cells, or perivascular nerve endings (see Table S3 for details). Subsequently, tissues were incubated for 1 h at room temperature with the secondary antibodies Alexa Fluor 555 and Alexa Fluor 488 (Table S3), processed using a TrueVIEW Autofluorescence Quenching Kit (Vector Laboratories, Newark, CA, USA) to reduce unwanted autofluorescence and background, then mounted in anti-fade mounting medium with DAPI (Vectashield, Vector Laboratories, Newark, CA, USA) to analyze the locations of the TRP channels using an ECHO Revolve Microscope (ECHO Laboratories, San Diego, CA, USA).

### 4.5. Measurements of CGRP Release in Organ Bath Fluid

Dermal artery segments were subjected to a similar protocol as that used during the functional studies (see Section 4.3). Bath fluids were collected in tubes containing aprotinin (0.6 TIU/mL) after creating the concentration–response curves from the segments treated with vehicles (DMSO for cinnamaldehyde and PregS or ethanol for capsaicin) or the agonists cinnamaldehyde, PregS, and capsaicin. Moreover, Krebs solution was used as a control and stored at −80 °C. A competitive radioimmunoassay (Peninsula Lab INC., San Carlos, CA, USA) was used according to the instructions of the manufacturer to measure the CGRP-LI levels in the bath fluid (detection range: 0.53–660 pmol/L), as previously reported [10].

### 4.6. Data Presentation and Statistical Analysis

All data in the text and figures are presented as mean ± SEM. The relaxant responses to the agonists were expressed as a percentage of relaxation of the tone induced by KCl 30 mM. The concentration–response curves were analyzed using nonlinear regression, and the potencies of the agonists were expressed as pEC_50_ using GraphPad Prism 8.0 (GraphPad Software, San Diego, CA, USA) (see Supplemental Methods, “S1.3 Calculation of pEC_50_”). The relaxation obtained at the highest agonist concentration was considered as the E_max_, and the differences between groups were evaluated by one-way ANOVA followed by Dunnett’s post hoc test. Moreover, the statistical differences between the concentration–response curves of the agonists in the absence and presence of the antagonists were evaluated with Dunnett’s post hoc test once a mixed-effects model had revealed that the samples represented different populations. Finally, CGRP levels in the bath fluids were analyzed using the non-parametric Kruskal–Wallis test, followed by Dunn’s post hoc multiple comparisons test [10]. Statistical significance was accepted at *p* < 0.05.

## 5. Conclusions

In human isolated dermal arteries obtained from pregnant women at the final stage of pregnancy, (i) the relaxation responses induced by cinnamaldehyde and capsaicin were not mediated by the activation of TRPA1 or TRPV1 channels, respectively, suggesting the involvement of non-specific mechanisms; and (ii) the vasodilatory responses induced by PregS were mediated by TRPM3 channels. Therefore, the regulation of vascular tone by TRPM3 channels can be attributed, at least in part, to CGRP-independent mechanisms, including the activation of NMDA receptors and/or the nitric oxide pathway.

## Data Availability

Data are contained within the article.

## Supplementary Materials

The following supporting information can be downloaded at: https://www.mdpi.com/article/10.3390/ph17020156/s1, Figure S1: Vasodilatory responses induced by TRP channel agonists in human isolated dermal arteries; Figure S2: Negative controls for immunofluorescence; Figure S3: Role of TRPM3 channels in the modulation of vascular tone; Table S1: Pharmacological compounds used in the present study; Table S2: pEC_50_ values of different compounds evaluated on the vasodilatory effect produced by TRP channel agonists in human isolated dermal arteries; Table S3: Primary and secondary antibodies used for immunofluorescence microscopy. References [10,52] are cited in the supplementary materials.
