# Supplementary material for: Functional Analysis of TRPA1, TRPM3, and TRPV1 Channels in Human Dermal Arteries and Their Role in Vascular Modulation
Source: Pharmaceuticals (Basel). 2024 Jan 25;17(2):156. doi: 10.3390/ph17020156 (PMC10892635; doi:10.3390/ph17020156)
Supplement: Supplementary file 1 [file pharmaceuticals-17-00156-s001.zip › pharmaceuticals-2814047-supplementary.pdf]

SUPPLEMENTAL MATERIALS

Functional Analysis of TRPA1, TRPM3, and TRPV1 Channels in Human Dermal Arteries and Their Role in Vascular Modulation

Eduardo Rivera-Mancilla [1], Linda Al-Hassany [1], Heleen Marynissen [2], Dorien Bamps [2], Ingrid M. Garrelds [1], Jérôme Cornette [3], A. H. Jan Danser [1], Carlos M. Villalón [4], Jan N. de Hoon [2] and Antoinette MaassenVanDenBrink [1]

[1] Division of Vascular Medicine and Pharmacology, Department of Internal Medicine, Erasmus University Medical Center Rotterdam, P.O. Box 2040, 3000 CA Rotterdam, The Netherlands
[2] Department of Pharmaceutical and Pharmacological Sciences, Center for Clinical Pharmacology, KU Leuven, 300 Leuven, Belgium
[3] Department of Obstetrics and Fetal Medicine, Erasmus University Medical Center Rotterdam, P.O. Box 2040, 3000 CA Rotterdam, The Netherlands
[4] Department of Pharmacobiology, Cinvestav-Coapa, Mexico City C.P. 14330, Mexico

S1. Supplementary methods

S1.1 Wire-myography experiments

All the dermal arteries were placed in a carbogenated Krebs bicarbonate buffer solution containing (mM): NaCl (118), KCl (4.7), $CaCl_2$ (2.5), $MgSO_4$ (1.2), KH_2PO_4 (1.2), $NaHCO_3$ (25) and glucose (8.3); pH 7.4, and suspended between two parallel stainless-steel wires (40-µm calibre) in 6 mL-organ baths filled with Krebs buffer at 37 °C and aerated with 95% O_2–5% CO_2. After equilibration for at least 30 min in the organ baths, with two changes of solution at 15 min intervals, blood vessel segments were subsequently stretched to a tension normalized to 90% of l_{100} (distance when transmural pressure equals 100 mmHg) [10,52]. After equilibration and normalized tension, the blood vessels were exposed to 30 mM KCl, while the maximum contractile response to KCl was determined using 100 mM KCl. After washout and stabilisation for at least 15 min, concentration-response curves were constructed. Data of these vessels were recorded using a LabChart data acquisition system (AD Instruments Ltd, Oxford, UK).

S1.2 Immunofluorescence microscopy

Intact dermal arteries were sectioned into 15-µm thick slices in a cryostat (Leica Biosystems, Wetzlar, Germany); and collected on adhesive microscope slides (Waldemar Knittel Glasbearbeitungs, Branuschweig, Germany). Mounted sections were fixed with 4% paraformaldehyde in phosphate buffer solution (PBS) during 10 min and permeabilized in PBS containing 0.2% Triton X-100 for 10 min. The tissue area in each microscope slide was delimited with a hydrophobic slide marker (Pap-Pen, Sigma-Aldrich, Germany). Then, tissues were blocked for 2 h with 150 µL of blocking buffer containing: PBS, 0.2% Triton X-100, 1% bovine serum albumin (BSA) and 10% normal donkey serum. After the blocking time, the slides were incubated overnight at 4°C with a primary antibody (diluted in PBS containing 0.2%

Triton X-100, 1% BSA and 1% normal donkey serum) against TRPA1, TRPM3 or TRPV1 channels in combination with the markers for endothelial cells, smooth muscle cells or perivascular nerve endings (see Table S3 for details). Subsequently, sections were incubated for 1 h at room temperature with the secondary antibodies Alexa Fluor 555 and Alexa Fluor 488 (Table S3) diluted in PBS containing 0.2% Triton X-100 and 1% BSA. Finally, sections were washed three times with PBS for 10 min each; processed with TrueVIEW Autofluorescence Quenching Kit (Vector Laboratories, Newark, CA, USA) to reduce unwanted autofluorescence and background following the manufacturer's instruction; and mounted in Anti-fade Mounting Medium with DAPI (Vectashield, Vector Laboratories, Newark, CA, USA). Negative controls were performed by omitting incubation of the tissue slices with the primary antibodies (see Figure S2). Fluorescence images were captured by using the ECHO Revolve Microscope (ECHO Laboratories, San Diego, CA, USA) processed with the software ImageJ (v1.49, National Institutes of Health, MD, USA) and Adobe Illustrator 2021 v26.0 (Adobe Inc., Mountain View, CA, USA)

S1.3 Calculation of pEC_{50}

The potency of the agonist was expressed as pEC_{50}, which was calculated using the GraphPad Prism 8.0 (GraphPad Software, San Diego, CA, USA). Considering that a plateau in the concentration−response curves was not reached, the response observed at the highest concentration of the agonists used (i.e., cinnamaldehyde: 1 mM; PregS: 100 μM; capsaicin: 100 μM; and CIM0216: 100 μM) was considered as E_{max}. It should be noted that E_{max} could have reached higher concentrations than those applied in our experimental protocols. Therefore, the pEC_{50} values of these concentration−response curves may be an overestimation of the actual pEC_{50} values. The statistical differences between pEC_{50} values were evaluated with Dunnet's post-hoc test, once an one−way ANOVA data revealed that the samples

represented different populations, followed by Dunn's post hoc multiple comparison test and the statistical significance was accepted at P≤0.05 [10].

S1.4 Compounds

The compounds used in the present study (obtained from the sources indicated) were: pregnenolone sulfate sodium salt (PregS, 3β-Hydroxy-5-pregnen-20-one 3-sulfate); cinnamaldehyde (3-Phenylprop-2-enal); capsaicin (8-Methyl-N-vanillyl-trans-6-nonenamide); capsazepine (9 N-[2-(4-Chlorophenyl)ethyl]-1,3,4,5-tetrahydro-7,8-dihydroxy-2H-2-benzazepine-2-carbothioamide); HC030031 (2-(1,3-dimethyl-2,6-dioxo-2,3,6,7-tetrahydro-1H-purin-7-yl)-N-[4-(propan-2-yl)phenyl]acetamide); indomethacin (1-(4-Chlorobenzoyl)-5-methoxy-2-methyl-3-indoleacetic acid); and Nitro-L-arginine methyl ester hydrochlorid (L-NAME, methyl (2S)-2-amino-5-(1-nitrocarbamimidamido)pentanoate) (Sigma-Aldrich, Schnelldorf, Germany); CIM 0216 (3,4-Dihydro-N-(5-methyl-3-isoxazolyl)-α-phenyl-1(2H)-quinolineacetamide); (+)-MK-801 maleate (5S,10R)-(+)-5-Methyl-10,11-dihydro-5H-dibenzo[a,d]cyclohepten-5,10-imine maleate); TRAM-34 (1-[(2-Chlorophenyl)diphenylmethyl]-1H-pyrazole); and apamin (Tocris-Bioscience, Bristol, UK); and isosakuranetin ((2S)-5,7-dihydroxy-2-(4-methoxyphenyl)-2,3-dihydrochromen-4-one) (MedChemExpress, Bio-Connect, Huissen, The Netherlands). When needed: (i) 70% ethanol was used to dissolve capsaicin; (ii) methanol to dissolve capsazepine; or (iii) a small amount of 1 N HCl to initially dissolve olcegepant, and then diluted with distilled water. The other compounds were dissolved in distilled water or DMSO. All compounds were stored in aliquots at -80°C.

Table S1. Pharmacological compounds used in the present study

Drug	Biological activity
Cinnamaldehyde	TRPA1 channel agonist
PregS	TRPM3 channel agonist *and modulator of NMDA receptors*
CIM0216	Synthetic TRPM3 channel agonist
Capsaicin	TRPV1 channel agonist
HC030031 (10 µM)	TRPA1 channel blocker
Isosakuranetin (5 µM)	TRPM3 channel blocker
Capsazepine (5 µM)	TRPV1 channel antagonist
Olcegepant (1 µM)	CGRP receptor antagonist
MK-801 (10 µM)	NMDA receptor antagonist
2−APB (75 µM)	TRPM3 channel antagonist
L−NAME (100 µM)	Nit6ric oxide synthase inhibitor
Indomethacin (0.1 µM)	Cyclooxygenase inhibitor
TRAM−34 (10 µM)	Blocker of intermediate-conductance Ca^{2+}-activated K^+ channels
Apamin (0.1 µM)	Blocker of small-conductance Ca^{2+}-activated K^+ channels

CGRP: calcitonin gene−related peptide; L−NAME: nitro−L-arginine methyl ester; NMDA: N−methyl−D−aspartate; PregS: pregnenolone sulfate; TRP: transient receptor potential (A: ankyrin; M: melastatin: V: vanilloid); 2−APB: 2−aminoethoxydiphenylborate

Table S2. pEC_{50} values of different compounds evaluated on the vasodilatory effect produced by TRP channel agonists in human isolated dermal arteries

**Treatment**	**pEC_{50}**
Cinnamaldehyde	
Control	3.57±0.33
HC030031 (10 µM)	3.63±0.32
Olcegepant (1 µM)	3.40±0.20
L−NAME (100 µM)	3.64±0.35
Indomethacin (0.1 µM)	3.65±0.31
TRAM−34 (10 µM) + Apamin (0.1 µM)	4.54±0.81
PregS	
Control	4.69±0.58
Isosakuranetin (5 µM)	4.81±0.54
Olcegepant (1 µM)	5.28±0.66
MK−801 (10 µM)	4.31±0.58
L−NAME (100 µM)	4.27±0.64
2−APB (75 µM)	4.89±0.20
Indomethacin (0.1 µM)	4.20±0.20
TRAM−34 (10 µM) + Apamin (0.1 µM)	4.05±0.20
Capsaicin	
Control	4.74±0.10
Capsazepine (5 µM)	4.70±0.40
Olcegepant (1 µM)	4.99±0.44
L−NAME (100 µM)	4.40±0.10
Indomethacin (0.1 µM)	4.50±0.13
TRAM−34 (10 µM) + Apamin (0.1 µM)	4.94±0.10
CIM 0216	
Control	5.64±2.20
Isosakuranetin (5 µM)	4.15±0.10

pEC_{50}: negative log of the molar concentration of the agonist needed to produce half of its maximal response.

Table S3. Primary and secondary antibodies used for immunofluorescence microscopy

Antibody	Catalog No.	Source	Dilution	Company
Primary antibodies				
TRPA1	OST00061W	Rabbit	1:800	Invitrogen, ThermoFisher Scientific
TRPM3	ACC-050	Rabbit	1:200	Alomone Labs
TRPV1	NB100-1617	Rabbit	1:600	Novus Biologicals
CD31, endothelial cells	M082301-2	Mouse	1:200	Agilent, Dako
Actin α−smooth muscle cells	A2547	Mouse	1:200	Sigma−Aldrich
PGP9.5	Ab8189	Mouse	1:100	Abcam
Secondary antibodies				
Alexa Fluor™ 488	A21206	Donkey	1:500	Invitrogen, ThermoFisher Scientific
Alexa Fluor 555	A31570	Donkey	1:500	Invitrogen, ThermoFisher Scientific

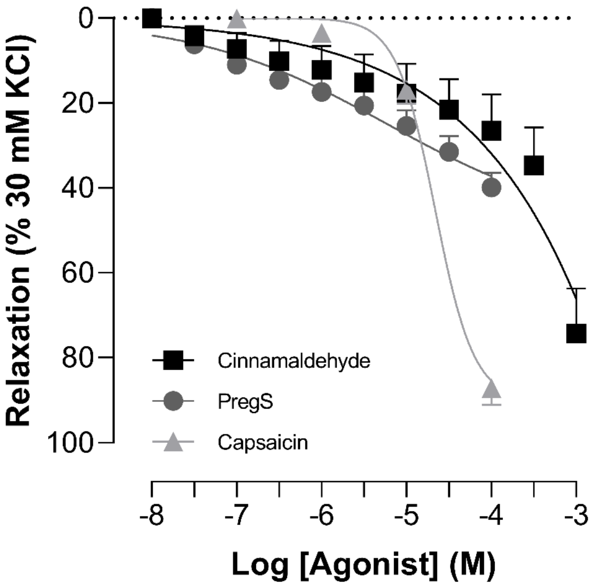

Figure S1. Vasodilatory responses induced by TRP channel agonists in human isolated dermal arteries. Relaxant responses capsaicin (0.1-100 µM, *n* = 19), cinnamaldehyde (0.01 µM–1 mM, *n* = 8) and PregS (0.01–100 µM, *n* = 22) in human isolated dermal arteries. Data are expressed as mean ± SEM. The control responses of the agonists represent the same data as those depicted in Figure 1 for comparative purposes.

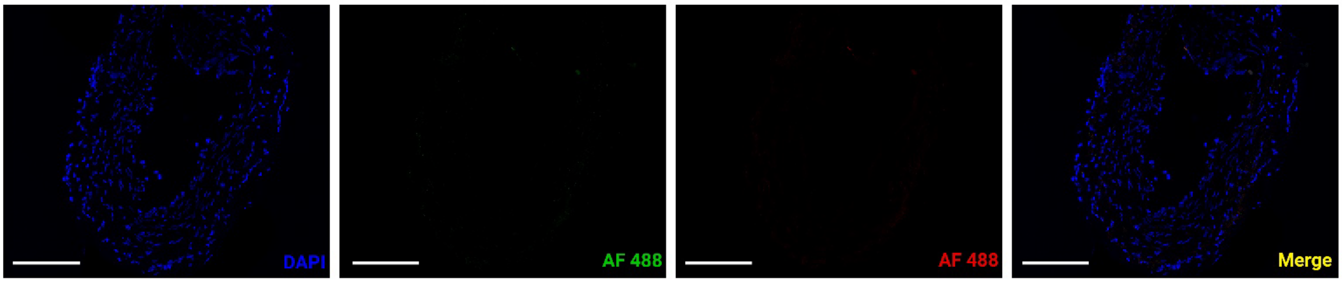

Figure S2. Negative controls for immunofluorescence. Confocal microscopy images of intact human dermal arteries labelled without the primary antibodies. Scale bars represent 170 μM. AF, Alexa Fluor.

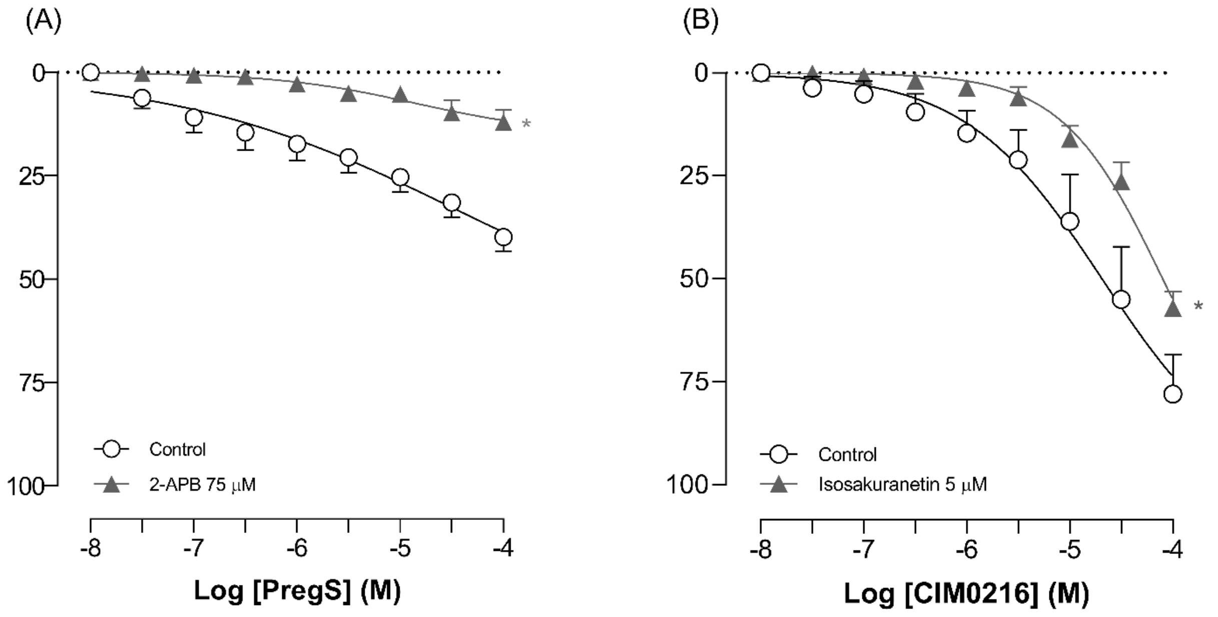

Figure S3. Role of TRPM3 channels in the modulation of vascular tone. Vasorelaxation induced by (A) PregS (0.01–100 µM, *n* = 8–22) (A) or (B) CIM 0216 (0.01–100 µM, *n* = 5) in the absence (control) or presence of (A) 2–APB (75 µM) or (B) isosakuranetin. The control response of the agonist in Figure S2A represents the same data as those shown in Figure 2B, but is shown here as the control for comparative purposes. Data are expressed as mean ± SEM. *P<0.05 *versus* control.
